# Supplementary material for: Women’s Media Use and Preferences of Media-Based Interventions on Lifestyle-Related Risk Factors in Gynecological and Obstetric Care: A Cross-Sectional Multi-Center Study in Germany
Source: Int J Environ Res Public Health. 2021 Sep 18;18(18):9840. doi: 10.3390/ijerph18189840 (PMC8466324; doi:10.3390/ijerph18189840)
Supplement: Supplementary file 1 [file ijerph-18-09840-s001.zip › ijerph-1352281-Supplementary.pdf]

Table S1. Questionnaire.

Liebe Teilnehmerin,

wir bitten Sie die nachfolgenden Fragen zu beantworten. Bitte beachten Sie, dass bei einigen Fragen Mehrfachnennungen möglich sind. Wenn Sie ein Kreuz an einer "falschen" Stelle gemacht haben und es ändern möchten, füllen Sie das falsche Kästchen bitte ganz aus und machen Sie Ihr Kreuzchen erneut. Die Fragen beziehen sich immer auf Ihre individuelle Meinung. Wenn Sie fertig sind, werfen Sie den Fragebogen bitte in die Box.

**1. Sind Sie momentan schwanger?**	
☐ Ja, ich bin in der ___ Schwangerschaftswoche.	☐ Nein → **Weiter mit Frage 3**
**2. War die Schwangerschaft geplant?**	
☐ Ja	☐ Nein
**3. Planen Sie innerhalb der nächsten 12 Monate schwanger zu werden?**	
☐ Ja → **Weiter mit Frage 5**	☐ Nein
**4. Planen Sie generell (wieder) schwanger zu werden?**	
☐ Ja	☐ Nein
**5. Planen Sie (wieder) zu stillen?**	
☐ Ja	☐ Nein
**6. Waren Sie schon einmal schwanger?**	
☐ Ja, das ist meine ___ Schwangerschaft.	☐ Nein → **Weiter mit Frage 9**
**7. Stillen Sie momentan?**	
☐ Ja	☐ Nein
**8. Haben Sie schon einmal gestillt?**	
☐ Ja	☐ Nein
**A Medien in der gynäkologischen Versorgung**	
**9. Welche der folgenden Medien mit Informationen zum Gesundheitsverhalten in der Schwangerschaft nehmen Sie bei Ihrer Frauenärztin/Ihrem Frauenarzt und/oder Ihrer Hebamme/Ihrem Entbindungspfleger wahr?** (Mehrfachnennungen sind möglich)	
**Frauenärztin/Frauenarzt:**	**Hebamme/Entbindungspfleger:**
☐ (Internet-) Adressen von Beratungs-/ Informationsstellen/ Portalen	☐ (Internet-) Adressen von Beratungs-/ Informationsstellen/ Portalen
☐ Apps	☐ Apps
☐ Bücher	☐ Bücher
☐ Flyer	☐ Flyer
☐ Informationsbroschüren	☐ Informationsbroschüren
☐ Merkzettel	☐ Merkzettel
☐ Tonaufnahmen/Podcasts	☐ Tonaufnahmen/Podcasts
☐ Video-/Filmmaterial	☐ Video-/Filmmaterial
☐ Zeitschriften	☐ Zeitschriften
☐ Keine Medien	☐ Keine Medien
☐ Sonstige Medien: ______________	☐ Sonstige Medien: ________________

**10. Welche der folgenden Medien mit Informationen zum Gesundheitsverhalten in der <u>Stillzeit</u> nehmen Sie bei Ihrer Frauenärztin/Ihrem Frauenarzt und /oder Ihrer Hebamme/Ihrem Entbindungspfleger wahr?** (Mehrfachnennungen sind möglich)

**Frauenärztin/Frauenarzt:**	**Hebamme/Entbindungspfleger:**
☐ (Internet-) Adressen von Beratungs-/ Informationsstellen/ Portalen	☐ (Internet-) Adressen von Beratungs-/ Informationsstellen/ Portalen
☐ Apps	☐ Apps
☐ Bücher	☐ Bücher
☐ Flyer	☐ Flyer
☐ Informationsbroschüren	☐ Informationsbroschüren
☐ Merkzettel	☐ Merkzettel
☐ Tonaufnahmen/Podcasts	☐ Tonaufnahmen/Podcasts
☐ Video-/Filmmaterial	☐ Video-/Filmmaterial
☐ Zeitschriften	☐ Zeitschriften
☐ Keine Medien	☐ Keine Medien
☐ Sonstige Medien: _______________	☐ Sonstige Medien: _________________

**11. Auf welche der folgenden Medien mit Informationen zum Gesundheitsverhalten in der <u>Schwangerschaft</u> wurden Sie von Ihrer Frauenärztin/Ihrem Frauenarzt und oder Ihrer Hebamme/Ihrem Entbindungspfleger hingewiesen?** (Mehrfachnennungen sind möglich)

**Frauenärztin/Frauenarzt:**	**Hebamme/Entbindungspfleger:**
☐ (Internet-) Adressen von Beratungs-/ Informationsstellen/ Portalen	☐ (Internet-) Adressen von Beratungs-/ Informationsstellen/ Portalen
☐ Apps	☐ Apps
☐ Bücher	☐ Bücher
☐ Flyer	☐ Flyer
☐ Informationsbroschüren	☐ Informationsbroschüren
☐ Merkzettel	☐ Merkzettel
☐ Tonaufnahmen/Podcasts	☐ Tonaufnahmen/Podcasts
☐ Video-/Filmmaterial	☐ Video-/Filmmaterial
☐ Zeitschriften	☐ Zeitschriften
☐ Keine Medien	☐ Keine Medien
☐ Sonstige Medien: _______________	☐ Sonstige Medien: _________________

**12. Auf welche der folgenden Medien mit Informationen zum Gesundheitsverhalten in der <u>Stillzeit</u> wurden Sie von Ihrer Frauenärztin/Ihrem Frauenarzt und oder Ihrer Hebamme/Ihrem Entbindungspfleger hingewiesen?** (Mehrfachnennungen sind möglich)

**Frauenärztin/Frauenarzt:**	**Hebamme/Entbindungspfleger:**
☐ (Internet-) Adressen von Beratungs-/ Informationsstellen/ Portalen	☐ (Internet-) Adressen von Beratungs-/ Informationsstellen/ Portalen
☐ Apps	☐ Apps
☐ Bücher	☐ Bücher
☐ Flyer	☐ Flyer
☐ Informationsbroschüren	☐ Informationsbroschüren
☐ Merkzettel	☐ Merkzettel
☐ Tonaufnahmen/Podcasts	☐ Tonaufnahmen/Podcasts
☐ Video-/Filmmaterial	☐ Video-/Filmmaterial
☐ Zeitschriften	☐ Zeitschriften
☐ Keine Medien	☐ Keine Medien
☐ Sonstige Medien: _______________	☐ Sonstige Medien: _________________

13. Verwendet Ihre Frauenärztin/Ihr Frauenarzt und/oder Ihre Hebamme/Ihr Entbindungspfleger Medien (Flyer, Videos, etc.) in der Aufklärung über das Gesundheitsverhalten in der Schwangerschaft?

Frauenärztin/ Frauenarzt:	Hebamme/Entbindungspfleger:
☐ Ja<br>☐ Nein → **Weiter mit Frage 15**	☐ Ja<br>☐ Nein → **Weiter mit Frage 15**

14. Welche der folgenden Medien werden von Ihrer Frauenärztin/Ihrem Frauenarzt und/oder Ihrer Hebamme/Ihrem Entbindungspfleger in der Aufklärung über das Gesundheitsverhalten in der Schwangerschaft verwendet? (Mehrfachnennungen sind möglich)

Frauenärztin/Frauenarzt:	Hebamme/Entbindungspfleger:
☐ (Internet-) Adressen von Beratungs-/ Informationsstellen/ Portalen<br>☐ Apps<br>☐ Bücher<br>☐ Flyer<br>☐ Informationsbroschüren<br>☐ Merkzettel<br>☐ Tonaufnahmen/Podcasts<br>☐ Video-/Filmmaterial<br>☐ Zeitschriften<br>☐ Keine Medien<br>☐ Sonstige Medien: _______________	☐ (Internet-) Adressen von Beratungs-/ Informationsstellen/ Portalen<br>☐ Apps<br>☐ Bücher<br>☐ Flyer<br>☐ Informationsbroschüren<br>☐ Merkzettel<br>☐ Tonaufnahmen/Podcasts<br>☐ Video-/Filmmaterial<br>☐ Zeitschriften<br>☐ Keine Medien<br>☐ Sonstige Medien: _________________

15. Verwendet Ihre Frauenärztin/Ihr Frauenarzt und/oder Ihre Hebamme/Ihr Entbindungspfleger Medien (Flyer, Videos, etc.) in der Aufklärung über das Gesundheitsverhalten in der Stillzeit?

Frauenärztin/ Frauenarzt:	Hebamme/Entbindungspfleger:
☐ Ja<br>☐ Nein → **Weiter mit Frage 17**	☐ Ja<br>☐ Nein → **Weiter mit Frage 17**

16. Welche der folgenden Medien werden von Ihrer Frauenärztin/Ihrem Frauenarzt und/oder Ihrer Hebamme/Ihrem Entbindungspfleger in der Aufklärung über das Gesundheitsverhalten in der Stillzeit verwendet? (Mehrfachnennungen sind möglich)

Frauenärztin/Frauenarzt:	Hebamme/Entbindungspfleger:
☐ (Internet-) Adressen von Beratungs-/ Informationsstellen/ Portalen<br>☐ Apps<br>☐ Bücher<br>☐ Flyer<br>☐ Informationsbroschüren<br>☐ Merkzettel<br>☐ Tonaufnahmen/Podcasts<br>☐ Video-/Filmmaterial<br>☐ Zeitschriften<br>☐ Keine Medien<br>☐ Sonstige Medien: _______________	☐ (Internet-) Adressen von Beratungs-/ Informationsstellen/ Portalen<br>☐ Apps<br>☐ Bücher<br>☐ Flyer<br>☐ Informationsbroschüren<br>☐ Merkzettel<br>☐ Tonaufnahmen/Podcasts<br>☐ Video-/Filmmaterial<br>☐ Zeitschriften<br>☐ Keine Medien<br>☐ Sonstige Medien: _________________

17. Wie gut fühlen Sie sich von Ihrer Frauenärztin/ Ihrem Frauenarzt und/oder Ihrer Hebammen/ Ihrem Entbindungspfleger über das Gesundheitsverhalten in der Schwangerschaft aufgeklärt?

Frauenärztin/ Frauenarzt:	Hebamme/Entbindungspfleger:
☐ sehr gut aufgeklärt<br>☐ gut aufgeklärt<br>☐ mittelmäßig aufgeklärt<br>☐ kaum aufgeklärt<br>☐ schlecht aufgeklärt<br>☐ gar nicht aufgeklärt	☐ sehr gut aufgeklärt<br>☐ gut aufgeklärt<br>☐ mittelmäßig aufgeklärt<br>☐ kaum aufgeklärt<br>☐ schlecht aufgeklärt<br>☐ gar nicht aufgeklärt

18. Wie gut fühlen Sie sich von Ihrer Frauenärztin/ Ihrem Frauenarzt und/oder Ihrer Hebammen/ Ihrem Entbindungspfleger über das Gesundheitsverhalten in der Stillzeit aufgeklärt?

**Frauenärztin/ Frauenarzt:**	**Hebamme/Entbindungspfleger**:
☐ sehr gut aufgeklärt	☐ sehr gut aufgeklärt
☐ gut aufgeklärt	☐ gut aufgeklärt
☐ mittelmäßig aufgeklärt	☐ mittelmäßig aufgeklärt
☐ kaum aufgeklärt	☐ kaum aufgeklärt
☐ schlecht aufgeklärt	☐ schlecht aufgeklärt
☐ gar nicht aufgeklärt	☐ gar nicht aufgeklärt

19. Stehen Ihnen Medien mit Informationen zum Gesundheitsverhalten in der Schwangerschaft und Stillzeit zur Verfügung?

**Für die Schwangerschaft:**	**Für die Stillzeit:**
☐ Ja	☐ Ja
☐ Nein → **Weiter mit Frage22**	☐ Nein → **Weiter mit Frage 22**

20. Nutzen Sie die Ihnen zur Verfügung stehenden Medien mit Informationen zum Gesundheitsverhalten in der Schwangerschaft und Stillzeit?

**Für die Schwangerschaft:**	**Für die Stillzeit:**
☐ Ja	☐ Ja
☐ Nein → **Weiter mit Frage 22**	☐ Nein → **Weiter mit Frage 22**

21. Welche der folgenden Medien mit Informationen zum gesundheitsrelevanten Verhalten nutzen Sie? (Mehrfachnennungen sind möglich)

**Über die Schwangerschaft:**	**Über die Stillzeit:**
☐ (Internet-) Adressen von Beratungs-/ Informationsstellen/ Portalen	☐ (Internet-) Adressen von Beratungs-/ Informationsstellen/ Portalen
☐ Apps	☐ Apps
☐ Bücher	☐ Bücher
☐ Flyer	☐ Flyer
☐ Informationsbroschüren	☐ Informationsbroschüren
☐ Merkzettel	☐ Merkzettel
☐ Tonaufnahmen/Podcasts	☐ Tonaufnahmen/Podcasts
☐ Video-/Filmmaterial	☐ Video-/Filmmaterial
☐ Zeitschriften	☐ Zeitschriften
☐ Keine Medien	☐ Keine Medien
☐ Sonstige Medien: _______________	☐ Sonstige Medien: _________________

B Zugang zu Medien

22. Wo befinden sich die Medien mit Informationen zum Gesundheitsverhalten in der Schwangerschaft und Stillzeit bei Ihrer Frauenärztin/Ihrem Frauenarzt und/oder Ihrer Hebamme/Ihrem Entbindungspfleger? (Mehrfachnennungen sind möglich)

**Frauenärztin/Frauenarzt:**	**Hebamme/Entbindungspfleger**:
☐ Im Wartebereich	☐ Im Wartebereich
☐ Bei der Anmeldung	☐ Bei der Anmeldung
☐ Im Eingangsbereich	☐ Im Eingangsbereich
☐ Im Behandlungsraum	☐ Im Behandlungsraum
☐ Keine Medien wahrgenommen	☐ Keine Medien wahrgenommen
☐ Ich weiß es nicht	☐ Ich weiß es nicht
☐ Anderer Ort:____________________	☐ Anderer Ort:_________________

23. Welche Hürden begegnen Ihnen beim Zugang zu diesen Medien? (Mehrfachnennungen sind möglich)

Medien mit Informationen zum Gesundheitsverhalten in der Schwangerschaft und Stillzeit sind…

☐ nicht frei zugänglich	☐ kostenpflichtig
☐ nur auf Nachfrage erhältlich	☐ nur vor Ort nutzbar
☐ nicht mit Hinweisen versehen	☐ nur digital
☐ nur analog	☐ nicht vorhanden
☐ nicht mit Hürden verbunden	☐ Sonstige Hürden: ________________
☐ aktuell nicht relevant für mich	

C Probleme in dem Umgang und der Nutzung mit/von Medien

24. Welche Probleme haben Sie in der Nutzung der zur Verfügung stehenden Medien?
(Mehrfachnennungen sind möglich)

Medien mit Informationen zum Gesundheitsverhalten in der Schwangerschaft und Stillzeit sind…

- ☐ schwer verständlich (Sprache)
- ☐ schwer verständlich (Inhalt)
- ☐ nicht informativ genug
- ☐ zu informativ
- ☐ von schlechter Qualität
- ☐ unproblematisch
- ☐ aktuell nicht relevant für mich
- ☐ nicht interessant
- ☐ zu zeitaufwändig
- ☐ kompliziert in der Handhabung
- ☐ nicht mit Erklärungen versehen
- ☐ nicht attraktiv
- ☐ sonstige Probleme: _______________

D Bevorzugte Medien

25. Wie wichtig finden Sie Informationen zur Aufklärung über ein empfohlenes Gesundheitsverhalten in der Schwangerschaft von den folgenden Informationsquellen?

a) Familie/Freunden/Bekannten
☐ sehr wichtig ☐ wichtig ☐ etwas wichtig ☐ kaum wichtig ☐ gar nicht wichtig

b) Ihrer Frauenärztin/Ihrem Frauenarzt
☐ sehr wichtig ☐ wichtig ☐ etwas wichtig ☐ kaum wichtig ☐ gar nicht wichtig

c) Ihrer Hebamme/Ihrem Entbindungspfleger
☐ sehr wichtig ☐ wichtig ☐ etwas wichtig ☐ kaum wichtig ☐ gar nicht wichtig

d) Medien, von Frauenärztinnen/Frauenärzten oder Hebammen/Entbindungspflegern erstellt:
☐ sehr wichtig ☐ wichtig ☐ etwas wichtig ☐ kaum wichtig ☐ gar nicht wichtig

e) **Medien, erstellt von Krankenkassen erstellt:**
☐ sehr wichtig ☐ wichtig ☐ etwas wichtig ☐ kaum wichtig ☐ gar nicht wichtig

f) **Medien, erstellt von Schwangerschafts-/Still-/Babyprodukte Herstellern etc.:**
☐ sehr wichtig ☐ wichtig ☐ etwas wichtig ☐ kaum wichtig ☐ gar nicht wichtig

26. Wie wichtig finden Sie Informationen zur Aufklärung über ein empfohlenes Gesundheitsverhalten in der Stillzeit von den folgenden Informationsquellen?

a) Familie/Freunden/Bekannten
☐ sehr wichtig ☐ wichtig ☐ etwas wichtig ☐ kaum wichtig ☐ gar nicht wichtig

b) Ihrer Frauenärztin/Ihrem Frauenarzt
☐ sehr wichtig ☐ wichtig ☐ etwas wichtig ☐ kaum wichtig ☐ gar nicht wichtig

c) Ihrer Hebamme/Ihrem Entbindungspfleger
☐ sehr wichtig ☐ wichtig ☐ etwas wichtig ☐ kaum wichtig ☐ gar nicht wichtig

d) Medien, von Frauenärztinnen/Frauenärzten oder Hebammen/Entbindungspflegern erstellt:
☐ sehr wichtig ☐ wichtig ☐ etwas wichtig ☐ kaum wichtig ☐ gar nicht wichtig

e) **Medien, erstellt von Krankenkassen erstellt:**
☐ sehr wichtig ☐ wichtig ☐ etwas wichtig ☐ kaum wichtig ☐ gar nicht wichtig

f) **Medien, erstellt von Schwangerschafts-/Still-/Babyprodukte Herstellern etc.:**
☐ sehr wichtig ☐ wichtig ☐ etwas wichtig ☐ kaum wichtig ☐ gar nicht wichtig

27. Welche Art an Medien mit Informationen zum Gesundheitsverhalten in der Schwangerschaft und Stillzeit wünschen Sie sich zur Nutzung vor Ort in einer Frauenarztpraxis und/oder Hebammenpraxis?

Frauenärztin/Frauenarzt:
- ☐ Analog (z.B. Buch, Zeitschrift, etc.)
- ☐ Digital (z.B. Film, App, etc.)
- ☐ Analog und digital
- ☐ Keine Medien

Hebamme/Entbindungspfleger:
- ☐ Analog (z.B. Buch, Zeitschrift, etc.)
- ☐ Digital (z.B. Film, App, etc.)
- ☐ Analog und digital
- ☐ Keine Medien

**28. Welche der folgenden Medien mit Informationen zum Gesundheitsverhalten in der Schwangerschaft und Stillzeit wünschen Sie sich, zur Nutzung <u>vor Ort</u> in der Frauenarztpraxis und/oder Hebammenpraxis?** (Mehrfachnennungen sind möglich)	
**<u>Frauenärztin/Frauenarzt:</u>**	**<u>Hebamme/Entbindungspfleger:</u>**
☐ (Internet-) Adressen von Beratungs-/ Informationsstellen/ Portalen	☐ (Internet-) Adressen von Beratungs-/ Informationsstellen/ Portalen
☐ Apps	☐ Apps
☐ Bücher	☐ Bücher
☐ Flyer	☐ Flyer
☐ Informationsbroschüren	☐ Informationsbroschüren
☐ Merkzettel	☐ Merkzettel
☐ Tonaufnahmen/Podcasts	☐ Tonaufnahmen/Podcasts
☐ Video-/Filmmaterial	☐ Video-/Filmmaterial
☐ Zeitschriften	☐ Zeitschriften
☐ Keine Medien	☐ Keine Medien
☐ Sonstige Medien: _______________	☐ Sonstige Medien: _________________

**29. Welche Art an Medien mit Informationen zum Gesundheitsverhalten in der Schwangerschaft und Stillzeit wünschen Sie sich, zur Nutzung <u>zu Hause</u>?**	
☐ Analog (z.B. Zeitschrift, Flyer, etc.)	☐ Digital (z.B. Film, Podcast, etc.)
☐ Analog <u>und</u> digital	☐ Keine Medien

**30. Welche der folgenden Medien mit Informationen zum Gesundheitsverhalten in der Schwangerschaft und Stillzeit wünschen Sie sich, zur Nutzung <u>zu Hause</u>?** (Mehrfachnennungen sind möglich)	
☐ (Internet-) Adressen von Beratungs-/ Informationsstellen/ Portalen	
☐ Apps	☐ Tonaufnahmen/Podcasts
☐ Bücher	☐ Video-/Filmmaterial
☐ Flyer	☐ Zeitschriften
☐ Informationsbroschüren	☐ Keine Medien
☐ Merkzettel	☐ Sonstige Medien:_______________

**31. Zu welchen der folgenden Themen haben Sie sich bereits Informationen eingeholt?** (Mehrfachnennungen sind möglich)		
Ernährung in der	☐ Schwangerschaft	☐ Stillzeit
Nahrungsergänzungsmittel in der	☐ Schwangerschaft	☐ Stillzeit
Koffein in der	☐ Schwangerschaft	☐ Stillzeit
Sport & Körperliche Aktivität in der	☐ Schwangerschaft	☐ Stillzeit
Alkohol/ Tabak/ illegale Drogen in der	☐ Schwangerschaft	☐ Stillzeit
Medikamente in der	☐ Schwangerschaft	☐ Stillzeit
Stress & Entspannungstechniken in der	☐ Schwangerschaft	☐ Stillzeit

**32. Zu welchen der folgenden Themen wünschen Sie sich mehr Informationen?** (Mehrfachnennungen sind möglich)		
Ernährung in der	☐ Schwangerschaft	☐ Stillzeit
Nahrungsergänzungsmittel in der	☐ Schwangerschaft	☐ Stillzeit
Koffein in der	☐ Schwangerschaft	☐ Stillzeit
Sport & Körperliche Aktivität in der	☐ Schwangerschaft	☐ Stillzeit
Alkohol/ Tabak/ illegale Drogen in der	☐ Schwangerschaft	☐ Stillzeit
Medikamente in der	☐ Schwangerschaft	☐ Stillzeit
Stress & Entspannungstechniken in der	☐ Schwangerschaft	☐ Stillzeit

**33. Welchen Schwerpunkt sollen die Informationen über die Schwangerschaft haben?** (Mehrfachnennungen sind möglich)	
Die Informationen über die Schwangerschaft sollen…<br>☐ Empfehlungen über einen gesunden Lebensstil/Verhalten (körperliche Aktivität, Ernährung, Stress, Suchtmittelkonsum) vermitteln.<br>☐ Empfehlungen zur Vermeidung eines risikobehafteten Lebensstils/ Verhaltens vermitteln.<br>☐ mögliche lebensstilbedingte Risiken in der Schwangerschaft vermitteln.<br>☐ Rezeptvorschläge für das Kochen im Alltag in der Schwangerschaft beinhalten.<br>☐ Bewegungsübungen für den Alltag in der Schwangerschaft beinhalten.<br>☐ Entspannungsübungen für den Alltag in der Schwangerschaft beinhalten.<br>☐ Vorschläge für Alternativen zu Suchtmitteln wie Tabak etc. beinhalten.<br>☐ Empfehlungen über zu vermeidende Medikamente beinhalten.<br>☐ Empfehlungen über notwendige Nahrungsergänzungsmitteln beinhalten.	
**34. Welchen Schwerpunkt sollen die Informationen über die Stillzeit haben?** (Mehrfachnennungen sind möglich)	
Die Informationen über die Stillzeit sollen…<br>☐ Empfehlungen über einen gesunden Lebensstil/Verhalten (körperliche Aktivität, Ernährung, Stress, Suchtmittelkonsum) vermitteln.<br>☐ Empfehlungen zur Vermeidung eines risikobehafteten Lebensstils/ Verhaltens vermitteln.<br>☐ mögliche lebensstilbedingte Risiken in der Stillzeit vermitteln.<br>☐ Rezeptvorschläge für das Kochen im Alltag in der Stillzeit beinhalten.<br>☐ Bewegungsübungen für den Alltag in der Stillzeit beinhalten.<br>☐ Entspannungsübungen für den Alltag in der Stillzeit beinhalten.<br>☐ Vorschläge für Alternativen zu Suchtmitteln wie Tabak etc. beinhalten.<br>☐ Empfehlungen über zu vermeidende Medikamente beinhalten.<br>☐ Empfehlungen über notwendige Nahrungsergänzungsmitteln beinhalten.	
**E Soziodemografische Daten**	
**35. Leben Sie derzeit in einer Partnerschaft?**	
☐ Ja	☐ Nein
**36. Wie alt sind Sie?** ________ Jahre	
**37. Wie viele leibliche Kinder haben Sie?** ________ leibliche Kinder	
**38. Was ist Ihr höchster erworbener Schulabschluss?**	
☐ Haupt- / Volksschulabschluss<br>☐ Abschluss Polytechnische Oberschule<br>☐ Abitur (Gymnasium bzw. EOS)<br>☐ Schule beendet ohne Schulabschluss	☐ Realschulabschluss (Mittlere Reife)<br>☐ Fachhochschulreife<br>☐ Anderer Schulabschluss:_________________<br>☐ (Noch) keinen Abschluss
**39. Haben Sie eine abgeschlossene berufliche/ akademische Ausbildung? Wenn ja, welche?**	
☐ Lehre (beruflich-betrieblich)<br>☐ Fachschule (Meister-/Technikerschule, Berufs-/Fachakademie)<br>☐ Diplom<br>☐ Promotion<br>☐ In beruflicher/akademischer Ausbildung (Studentin/Auszubildende)<br>☐ Anderer Ausbildungsabschluss:___________________	☐ Berufs-/ Handelsschule (beruflich-schulisch)<br>☐ Fachhoch-/Ingenieurschule<br>☐ Bachelor<br>☐ Master/ Magister/ Staatsexamen<br>☐ Habilitation<br>☐ Kein beruflicher/akademischer Abschluss (und auch nicht in der Ausbildung)

40. In welcher beruflichen Stellung sind/waren Sie hauptsächlich beschäftigt?

Arbeiterin:

- ☐ Ungelernte Arbeiterin
- ☐ Angelernte Arbeiterin
- ☐ Gelernte Arbeiterin/ Facharbeiterin
- ☐ Vorarbeiterin, Meisterin, o.ä.

Selbstständige (einschließlich mithelfender Familienmitglieder):

- ☐ Landwirtin/ Genossenschaftsbäuerin
- ☐ Akademikerin/ freier Beruf
- ☐ Sonstiges mit bis zu 9 Mitarbeitern
- ☐ Sonstiges mit 10 und mehr Mitarbeitern
- ☐ Mithelfende Familienangehörige

Angestellte:

- ☐ Industrie-/ Werkmeisterin
- ☐ Einfache Tätigkeit (z.B. Verkäuferin)
- ☐ Qualifizierte Tätigkeit (z.B. Buchhalterin, Sachbearbeiterin, o.ä.)
- ☐ Hochqualifizierte Tätigkeit/ Leitungsfunktion (z.B. Abteilungsleiterin, Prokuristin, o.ä.)
- ☐ Umfassende Führungsaufgaben (z.B. Geschäftsführerin, Direktorin, o.ä.)

Beamtin (einschließlich Richterin, Berufssoldatin):

- ☐ Einfacher Dienst
- ☐ Mittlerer Dienst
- ☐ Gehobener Dienst
- ☐ Höherer Dienst

Sonstige:

- ☐ Schülerin, Studentin, Praktikantin, Auszubildende o.ä.
- ☐ Hausfrau

41. In welcher Krankenversicherung sind Sie versichert? (Mehrfachnennungen sind möglich)

Gesetzliche Krankenversicherung

- ☐ Allgemeine Ortskrankenkasse
- ☐ Ersatzkrankenkasse (Barmer, DAK, TK, GEK, usw.)
- ☐ Betriebskrankenkasse (BKK)
- ☐ Innungskrankenkasse (IKK)
- ☐ Andere gesetzliche Krankenkassen

Private Krankenversicherung

- ☐ Vollversicherung
- ☐ Zusatzversicherung

Andere Krankenversicherung

- ☐ Beihilfe (z.B. Öffentlicher Dienst)
- ☐ Ausländische Krankenkasse
- ☐ Sonstiger Anspruch auf Krankenversorgung

☐ Keine Krankenversicherung

42. Wie hoch ist das durchschnittliche monatliche Haushaltseinkommen, d.h. das Nettoeinkommen, das alle Haushaltsmitglieder zusammen nach Abzug von Steuern und Sozialabgaben haben? (Einschließlich Erziehungsgeld und Kindergeld)

- ☐ unter 500 Euro
- ☐ 500 bis unter 750 Euro
- ☐ 750 bis unter 1.000 Euro
- ☐ 1.000 bis unter 1.250 Euro
- ☐ 1.250 bis unter 1.500 Euro
- ☐ 1.500 bis unter 1.750 Euro
- ☐ 1.750 bis unter 2.000 Euro
- ☐ 2.000 bis unter 2.250 Euro
- ☐ 2.250 bis unter 2.500 Euro
- ☐ 2.500 bis unter 3.000 Euro
- ☐ 3.000 bis unter 4.000 Euro
- ☐ 4.000 bis unter 5.000 Euro
- ☐ 5.000 Euro und mehr

43. In welchem Land sind Sie geboren?

- ☐ Deutschland
- ☐ Anderes Land:________________________

44. In welchem Land sind Ihre Eltern geboren?

Mutter:

- ☐ Deutschland
- ☐ Anderes Land:____________________

Vater:

- ☐ Deutschland
- ☐ Anderes Land:____________________

Vielen herzlichen Dank für Ihre Teilnahme!
